# The Composition of Placental Microbiota and Its Association With Adverse Pregnancy Outcomes

**DOI:** 10.3389/fmicb.2022.911852

**Published:** 2022-07-18

**Authors:** Xuena La, Yuezhu Wang, Xu Xiong, Liandi Shen, Weiyi Chen, Lifeng Zhang, Fengyun Yang, Xushan Cai, Huajun Zheng, Hong Jiang

**Affiliations:** ^1^School of Public Health, Key Lab of Health Technology Assessment, National Health Commission of the People's Republic of China, Fudan University, Shanghai, China; ^2^NHC Key Lab of Reproduction Regulation, Shanghai Institute for Biomedical and Pharmaceutical Technologies, Fudan University, Shanghai, China; ^3^Shanghai-MOST Key Laboratory of Health and Disease Genomics, Chinese National Human Genome Center at Shanghai (CHGC) and Shanghai Institute for Biomedical and Pharmaceutical Technologies (SIBPT), Shanghai, China; ^4^School of Public Health and Tropical Medicine, Tulane University, New Orleans, LA, United States; ^5^Department of Administrative office, Shanghai Jiading Maternal and Child Health Hospital, Shanghai, China; ^6^Department of Clinical Laboratory, Shanghai Jiading Maternal and Child Health Hospital, Shanghai, China

**Keywords:** placental microbiota, adverse pregnancy outcomes, gestational diabetes mellitus, premature rupture of membranes, 16S rRNA

## Abstract

To verify whether the placenta harbors bacteria, and to explore the composition of placental microbiota (if yes) and its association with adverse pregnancy outcomes. The placental microbiota was detected by 16S rRNA gene sequencing technology. In the process of detecting placental samples, exogenous marine bacterial DNA that does not exist in the human body was artificially added to obtain a visible 16S band. At the same time, the sterile samples, such as scissors, sheets, and cotton swabs, in delivery and operating rooms were collected as the environmental control samples. As a result, a total of 2,621,009 sequences were obtained from 71 samples, 88.9% of which came from artificially added exogenous bacterial DNA, suggesting that the placenta contained fewer bacteria. After removing the operational taxonomic units (OTUs) that coexisted in environmental controls, the placenta was annotated with 11 phyla, 22 classes, 43 orders, 79 families, and 157 genera. The β diversity analysis showed that there were significant differences in the placental microbiota between 10 women with gestational diabetes mellitus (GDM) (*p*_AMOVA_ = 0.01) or 19 women with premature rupture of membranes (PROM) (*p*_AMOVA_ = 0.004), and 21 women without adverse pregnancy outcomes, respectively. There were higher abundances of genera *Bifidobacterium, Duncaniella*, and *Ruminococcus* in the placenta samples of women with GDM. The genera of *Bacteroides, Paraprevotella*, and *Ruminococcus* were more enriched in the placental samples of women with PROM. The authors concluded that the placenta may harbor small amounts of microbiota, and significant differences in the dominant microbiota of the placenta were observed between those pregnant women with and without adverse pregnancy outcomes.

## Introduction

For a long time, the placenta has been considered a completely sterile environment. However, the paradigm of “intrauterine sterility” has been challenged in recent decades, especially since the development of high-throughput sequencing technology and its application in placenta microbiota research (Aagaard et al., 2014; Collado et al., 2016; Zheng et al., 2017a). In 2014, Aagaard et al. sequenced 320 placental samples through 16S rRNA gene and whole-genome shotgun (WGS) sequencing and revealed the unique microbiota of placenta composed of non-pathogenic bacteria, such as phyla *Firmicutes, Tenericutes, Proteobacteria, Bacteroidetes*, and *Fusobacteria* (Aagaard et al., 2014). Parnell et al. used 16S rRNA sequencing technology to detect different areas of the placenta, such as basal plate, placental villous, and fetal membrane, and believed that the placental microbiota has niche-specificity (Parnell et al., 2017). Moreover, based on metagenomic sequencing, a study found that the placentas of preterm infants with severe chorioamnionitis had a higher abundance of *Ureaplasma parvum, Fusobacterium nucleatum*, and *Streptococcus agalactiae* (Prince et al., 2016). Zheng et al. disclosed that the placentas of fetal macrosomia are significantly enriched with *Proteobacteria, Firmicutes*, and *Gemmatimonadetes* compared with normal birth weight newborns (Zheng et al., 2017a). Other studies indicated that the features of placental microbiota can predict adverse pregnancy outcomes, such as preeclampsia and excessive gestational weight gain (Barak et al., 2007; Antony et al., 2015).

However, whether the bacteria existed in the placenta is debatable. Leiby et al. quantified absolute amounts of bacterial 16S rRNA gene sequences in the placental samples and found that the levels were as low as negative controls (Leiby et al., 2018). Another study also concluded that the fetal *in utero* environment was sterile using several microbiological and molecular methods in parallel, such as 16S rRNA gene amplification, RT-qPCR, Gram stain, immunohistochemistry for bacteria, and electron microscopy (Kuperman et al., 2020). Using a variety of rigorous DNA extraction and detection methods, De Goffau et al. detected the placental tissues of 537 parturient women, including 318 with adverse pregnancy outcomes and 219 healthy controls and found that the extractable bacterial sequence biomass in the placenta was extremely low, with almost only *Streptococcus agalactiae* detectable in 5% of placental samples. The study believed that there was no specific microbiome in the human placenta, but there might be a pathogenic bacterial infection in the placenta (De Goffau et al., 2019).

To further validate the existence of microbiota in the placenta, it is critical to use the highly sensitive technique to detect low amounts of bacteria and avoid possible environmental contamination. The purposes of this study were to (1) detect whether the placenta harbors microbiota and what were the characteristics of microbiota composition if bacteria were detectable in placentas, and (2) explore whether the characteristics of the placental microbiota were associated with adverse pregnancy outcomes.

## Materials and Methods

### Study Population

The study was based on the Fudan Preconceptional Offspring Trajectory Study (PLOTS) (Harville et al., 2019). Women were recruited when they attended the preconception health checkups in the preconception care clinic of Maternal and Child Health Hospital of Jiading District, Shanghai. Preconception women were eligible for the cohort if they (1) had intention to conception at the recruitment; (2) were aged 20–49 years; and (3) were willing to be followed through pregnancy until childbirth. The participants were excluded if they withdrew from the cohort before childbirth.

A self-administered questionnaire survey was carried out to collect women's demographic information after preconception baseline recruitment. Information about pregnancy complications and mode of delivery was extracted from the medical records.

This study was approved by the Ethics Committee of the School of Public Health, Fudan University (IRB#2016-10-0601, IRB#2019-07-0770, IRB#2020-01-0794), and all research participants provided written informed consent before the recruitment.

### Biological Sample Collection

A total of 61 placental samples were tested in this study. The placental samples were collected by trained midwives and nurses in a sterile environment of the operating and delivery rooms from February 2019 to July 2020. Sterile scissors were used to take a 1 × 1 cm piece of the placenta from the maternal side at 3, 6, 9, and 12:00 and the center point of the placenta, respectively. These pieces were immediately put in a sterile cryopreservation tube and placed in a −80°C temperature refrigerator.

In addition, 10 environmental control samples were collected, five from the delivery room and five from the operating room. In the delivery room, two sterile cotton swabs were collected after repeatedly wiping the surface with sterile scissors. Two pieces of the fabric sheet where the placenta was placed were also obtained. Furthermore, one sterile cotton swab was placed in the air for 10 min and then collected as the blank environment control sample. Other five environmental samples were also collected in the operating room using the same methods. All samples were placed in 15 ml sterile centrifuge tubes and immediately stored in an ultralow temperature refrigerator at −80°C.

### 16S rRNA Gene High-Throughput Sequencing

The QIAampPowerFecal Pro DNA Kit (Qiagen, USA) was used for DNA extraction following the manufacturer's protocol. Placental tissue samples were grounded with a tissue homogenizer. Nonwoven fabric cloths and cotton swabs were placed in PowerBead Pro Tubes, soaked, and shaken with Solution CD1 for 10 min.

For the detection of the bacterial 16S rRNA gene, polymerase chain reaction (PCR) amplification of the V3-V4 region was performed using the barcode-specific primers 338F (5′-ACTCCTACGGGAGGCAGCAG) and 806R (5′-GGACTACHVGGGTWTCTAAT). PCR with nuclease free water as template was set as a negative control. Due to the extremely low bacterial content of the placental samples, most of the extracted DNA could not be amplified to produce visible electrophoresis bands. Therefore, the genomic DNA of a marine bacterium *Loktanella* sp., which does not exist in humans, was artificially added to the DNA of placental and control samples (0.01 ng exogenous DNA for each sample). All amplicons were purified with a QIAquick PCR Purification Kit (Qiagen, USA) and pooled with equal concentrations. Then, the pooled amplicons were sequenced on an Illumina MiSeq instrument with a 2 × 300 cycles.

The sequence data have been deposited in the National Omics Data Encyclopedia (NODE, https://www.biosino.org/node/) under accession number OEP003119.

### Definition of Pregnancy Outcomes

Two adverse pregnancy outcomes—gestational diabetes mellitus (GDM) and premature rupture of membranes (PROM) were selected for exploring the association between placenta microbiota and pregnancy outcomes. GDM was diagnosed by the Oral Glucose Tolerance Test (OGTT) performed at 24–28 gestational weeks in the morning, after at least an 8 h-overnight fast, when the fasting plasma glucose was ≥5.1 mmol/L and/or 1 h post-OGTT glycemia ≥ 10.0 mmol/L and/or 2 h post-OGTT glycemia ≥ 8.5 mmol/L, according to international criteria (Hod et al., 2015).

Premature rupture of membrane was defined as the rupture of the fetal membranes before the onset of labor (Committee on Practice Bulletins-Obstetrics, 2018).

### Bioinformatics Analysis and Statistical Analysis Methods

Then, Quantitative Insights Into Microbial Ecology 2 (QIIME2, 2021.11) (Bolyen et al., 2019) and R 3.6.3 (package: amplicon, vegan) were used to analyze the microbiota data. The LefSe1.0 (LDA Effect Size) online analysis tool (Segata et al., 2011) was used to identify the iconic oral bacteria in different groups using Linear Discriminant Analysis (LDA) effect value method. The threshold of the logarithmic LDA score for discriminative features was 2.0. Taxonomy assignment was performed by BLASTN against the NCBI database and SILVA-132 database. Principal coordinate analysis (PcoA) was based on the Bray-Curtis distance matrix and analysis of molecular variance (AMOVA) was used to analyze the effect of different subgroup factors on the β diversity of placental microbiota.

## Results

### Characteristics of Participants

A total of 61 pregnant women with placental samples were included in the study. The mean age of women at preconception recruitment was 27 (range: 23–38) years old. The median month from preconception to conception was 3.28 (SD 3.16). The mean gestation age at childbirth was 39.17 (range: 36.14–41.57) weeks. In total, nineteen women were multipara (31.1%, 19/61). More than half of them (67.2%, 41/61) had college or above degree. The majority of these participants (73.8%, 45/61) had an annual family income of over 100,000 yuan (~15,720 USD). Among a total of 61 pregnant women, 10 women had GDM, 19 had PROM, one woman complicated from both PROM and GDM, eleven women had other pregnancy complications (three with anemia, two with microsomia, one with arrythmia, one with intrahepatic cholestasis of pregnancy, one with hypothyroidism, one with condyloma acuminatum, and one with fetal distress), and 21 women had no adverse pregnancy outcomes.

### Characteristics of Placental Microbiota

A total of 2,621,009 sequences were obtained from 71 samples, including 61 placental samples and 10 environmental control samples. Among them, 88.9% sequences (2,329,519/2,621,009) were artificially added exogenous bacterial DNA, indicating low bacterial content in the placental samples.

**Figure 1:**
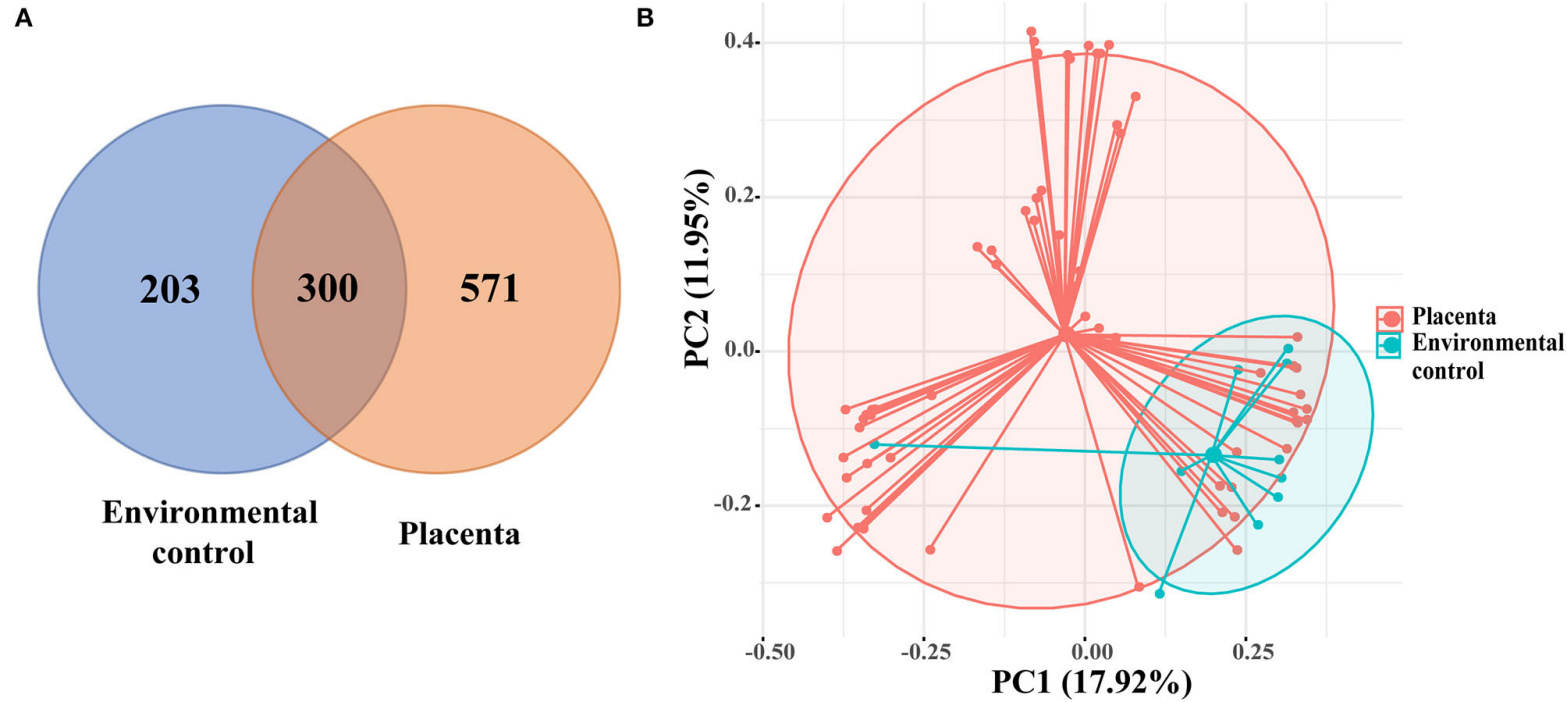
**(A)** The Venn diagram showed the shared operational taxonomic units (OTUs) among placental and environmental control samples. **(B)** Principal coordinate analysis (PCoA) analysis of the microbiota between placental and environmental control samples based on the Bray-Curtis distance matrix. Each point corresponds to a sample colored by the group (placenta and environmental control).

After excluding the exogenous bacterial DNA and non-16S DNA sequences, a total of 1,074 OTUs were revealed, with 300 OTUs shared by 61 placental samples and 10 environment control samples. Other 571 OTUs are presented in the placenta samples (Figure 1A). The PCoA showed the composition of placental microbiota overlapped with the environmental control samples based on the Bray-Curtis distance matrix (Figure 1B), and a significant difference was found in microbiota structure between the placenta and the environmental control *via* AMOVA analysis (*p* = 0.003).

**Figure 2:**
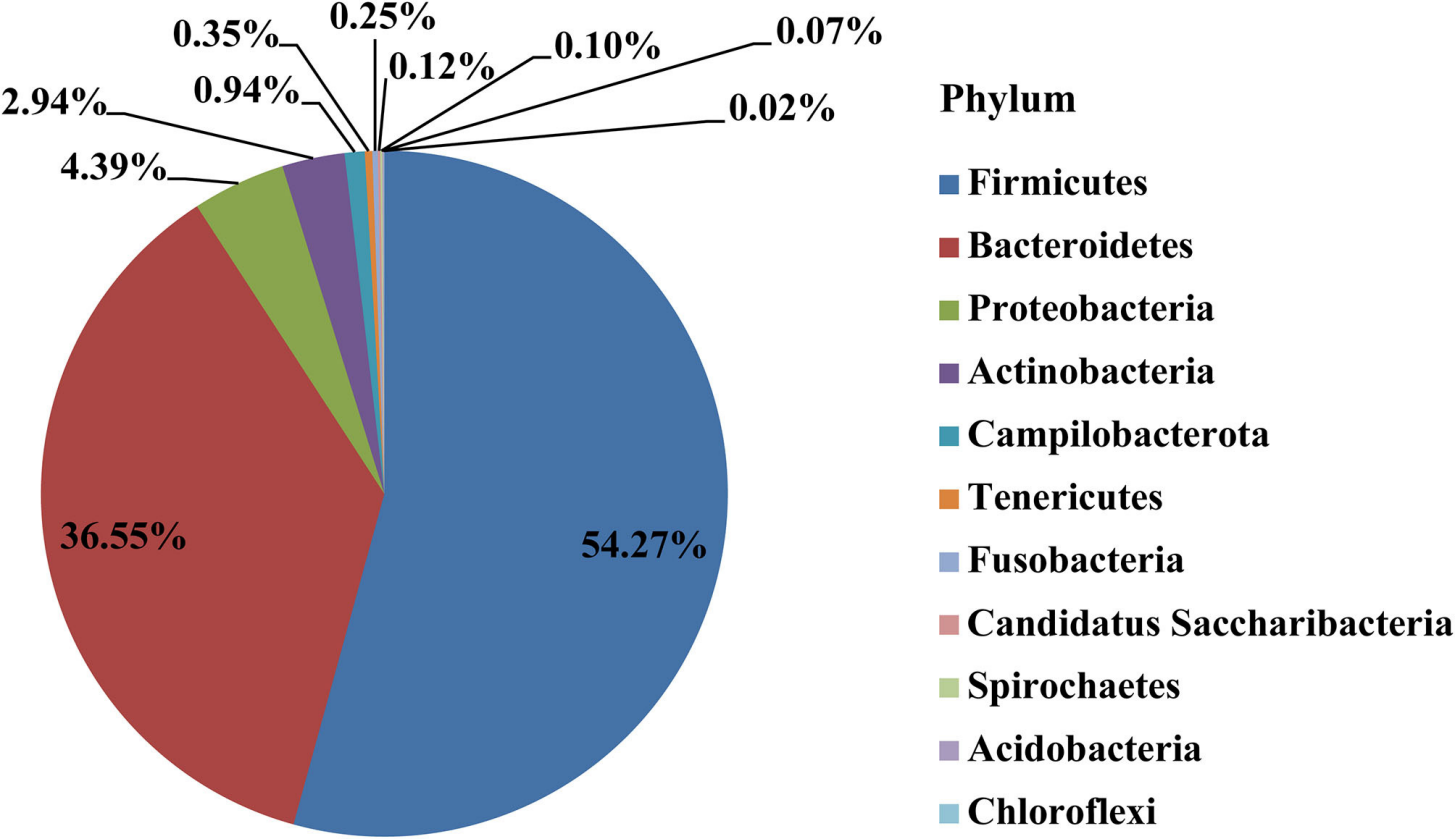
The distribution of placental microbiota at the phylum level.

After removing the 300 OTUs existing in environmental control samples (representing potentially contaminating OTU data), a total of 11 phyla, 22 classes, 43 orders, 79 families, and 157 genera were annotated in the remaining 571 OTUs of placental samples. The main dominant phyla in the placenta were *Firmicutes* (54.27%), *Bacteroidetes* (36.55%), *Proteobacteria* (4.39%), *Actinobacteria* (2.94%), etc. (Figure 2).

At genus level, 16 dominant genera (> 0.5%) accounted for 30.19% of all placenta microbiota (Table 1), but none was revealed in all 61 placental samples. The most abundant genus, *Alistipes*, was revealed in 39 samples, while the genus *Duncaniella* existed in 32 placental samples.

**Table 1:** The relative abundance of dominant genera in the placenta samples.

**Genus**	**Proportion**	**Number of Samples**
*Duncaniella*	7.29%	32
*Alistipes*	5.30%	39
*Odoribacter*	2.28%	12
*Prevotella*	2.09%	20
*Bacteroides*	2.06%	21
*Bifidobacterium*	2.03%	13
*Parasutterella*	1.62%	12
*Limosilactobacillus*	1.50%	5
*Ruminococcus*	1.14%	16
*Anaerotignum*	0.96%	23
*Helicobacter*	0.83%	14
*Paraprevotella*	0.72%	5
*Eisenbergiella*	0.63%	10
*Dialister*	0.61%	17
*Flavonifractor*	0.57%	3
*Intestinimonas*	0.55%	12

### Placenta Microbiota and Adverse Pregnancy Outcomes

**Figure 3:**
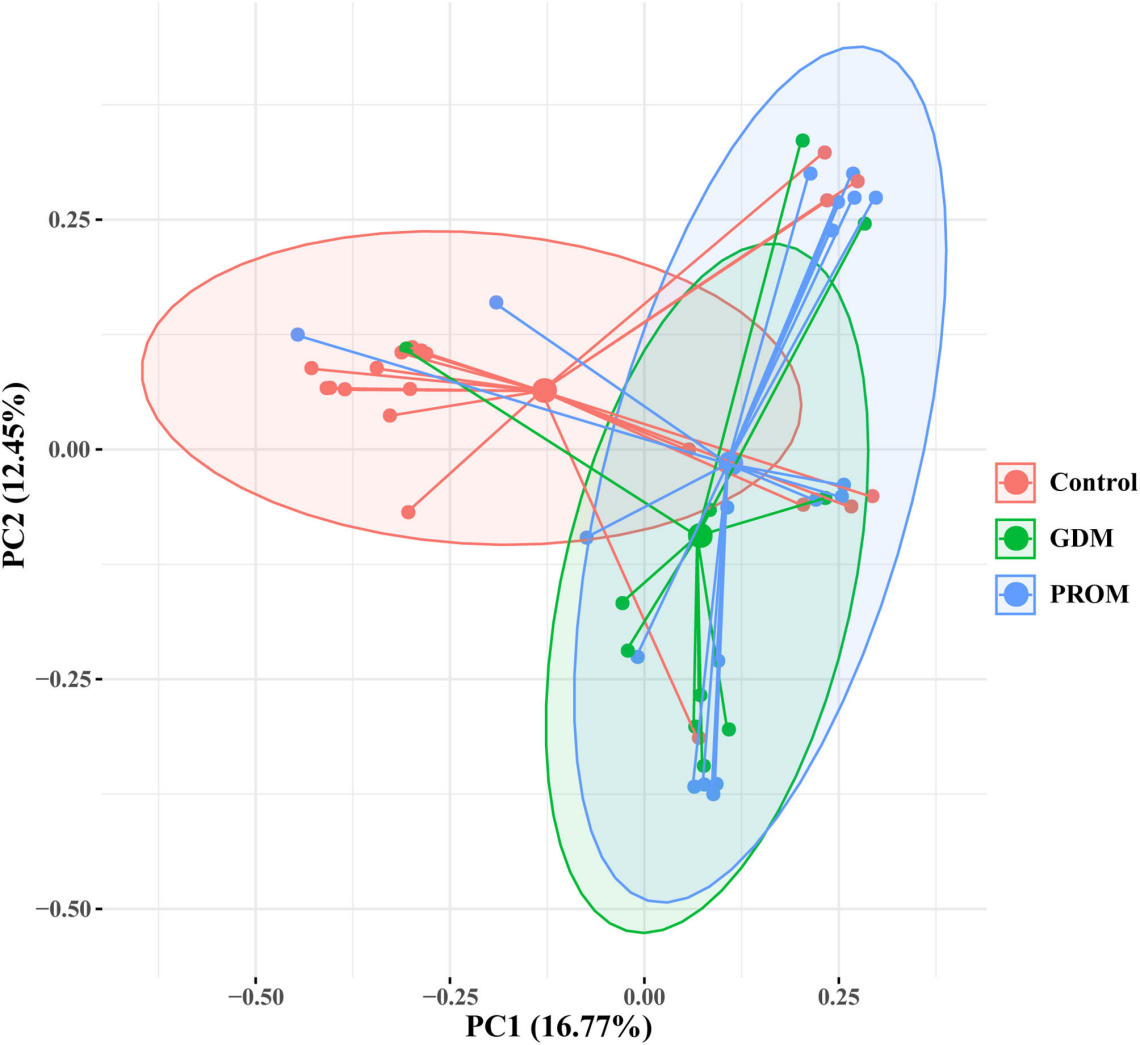
Principal coordinate analysis of placental microbiota with different pregnancy outcomes based on the Bray-Curtis distance matrix. Each point corresponds to a sample colored by the group (healthy control, GDM, and PROM).

The placental microbiota of women without adverse pregnancy outcomes showed significant difference with the combined samples of GDM (*p*_AMOVA_ = 0.01) and PROM (*p*_AMOVA_ = 0.004) (Figure 3). While there was no significant difference in placental microbiota composition between the groups with GDM and PROM (*p*_AMOVA_ = 0.407).

**Figure 4:**
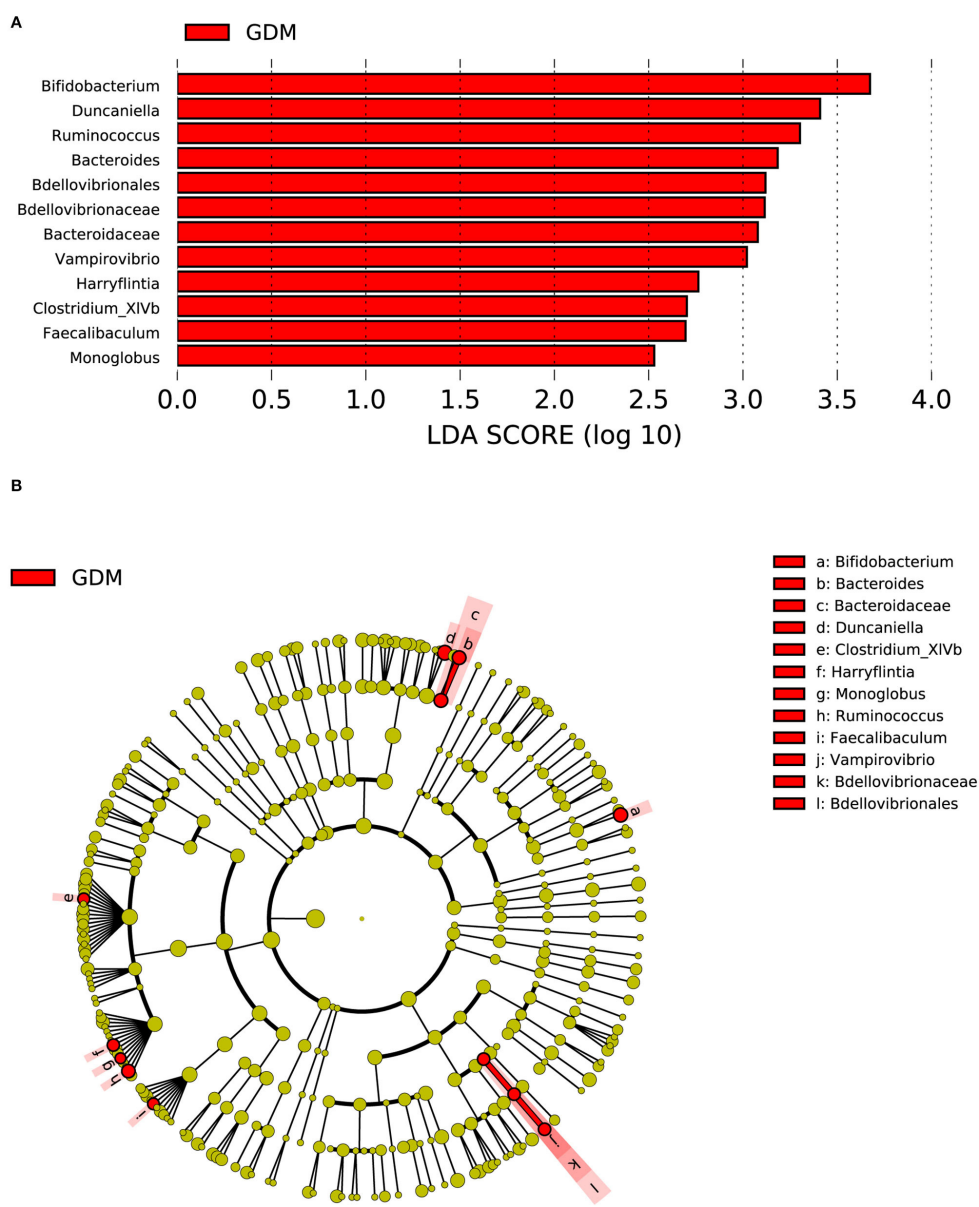
Distinct bacterial taxa for the placental microbiota between the group with GDM and the healthy control group was identified by the LEfSe analysis. **(A)** A histogram of the linear discriminant analysis (LDA) scores represents significant differences in the abundance of the bacterial taxa between the GDM group and the healthy control group. **(B)** A cladogram for taxonomic representation representing distinct bacterial taxa between the two groups. Red color indicates enrichment in the GDM samples. The diameter of each circle is proportional to the taxon's abundance.

Compared with the group of 21 women without adverse pregnancy outcomes, one order (*Bdellovibrionales*), two families (*Bacteroidaceae* and *Bdellovibrionaceae*), and nine genera (*Bifidobacterium, Duncaniella*, etc.) were significantly enriched in the placenta of the group consisting of 10 women with GDM (Figure 4).

**Figure 5:**
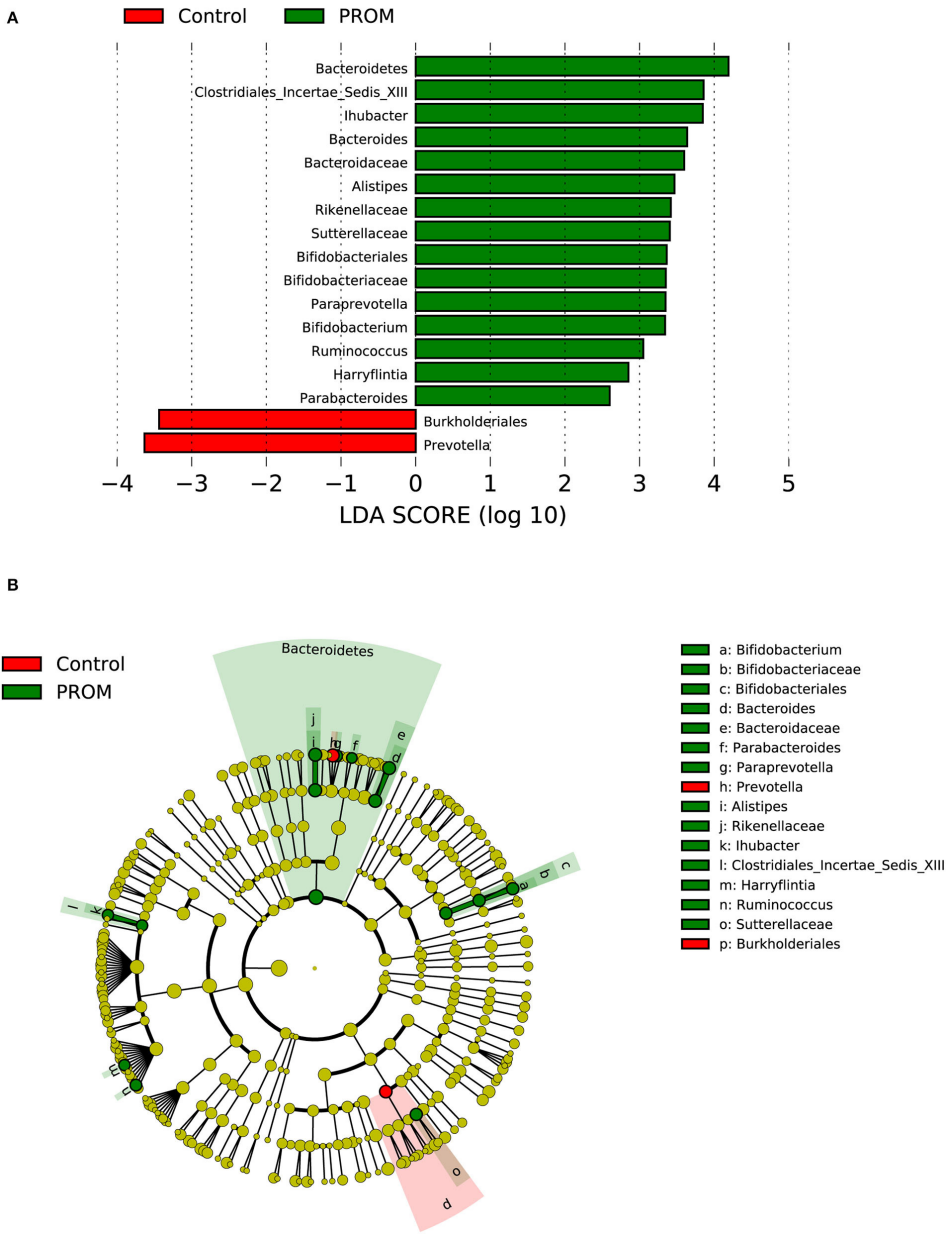
Distinct bacterial taxa for the placental microbiota between the PROM group and the healthy control group was identified by the LEfSe analysis. **(A)** A histogram of the linear discriminant analysis (LDA) scores represents significant differences in the abundance of the bacterial taxa between the PROM group and the healthy control group. **(B)** A cladogram taxonomic representation representing distinct bacterial taxa between the two groups. Green color indicates enrichment in the PROM group, and red indicates enrichment in the healthy control samples. The diameter of each circle is proportional to the taxon's abundance.

In the PROM group containing 19 cases (Figure 5), one phylum (*Bacteroidetes*), one order (*Bifidobacteriales*), five families (*Bacteroidaceae, Bifidobacteriaceae, Clostridiales_Incertae_Sedis_XIII, Rikenellaceae, and Sutterellaceae*), and eight genera (*Alistipes, Ihubacter*, etc.) were enriched in the placenta. *Burkholderiales* at the order level and *Prevotella* at the genus level were significantly decreased compared with placental samples from 21 women without adverse pregnancy outcomes.

Furthermore, the genera *Bacteroides, Bifidobacterium, Ruminococcus*, and *Harryflintia* were all significantly increased in both groups of GDM and PROM, compared with the healthy control groups.

## Discussion

To date, there are still big controversies regarding the existence of placental microbiota. In this study, during our pre-test, no visible electrophoresis bands were amplified from placental DNA, which indicated that the biomass of placenta microbiota was very low even if there was microbiota in placenta. Therefore, we added the DNA of a marine bacterium, *Loktanella* sp., into placental DNA to help library construction. After removing this artificially introduced DNA sequences and OTUs existed in environmental blank control samples, we obtained 571 OTUs that only existed in the placenta. Though OTUs were observed in each of the 61 placental samples, we did not find a “common/ shared” OTU in all placental samples. The two most abundant genera *Alistipes* and *Duncaniella* were separately revealed in 39 and 32 placental samples, indicating that placental microbiota were in low amount and differential among pregnant women. This may suggest that there were no common bacteria that existed in all the placental samples.

In 2014, Aagaard et al. used high-throughput sequencing technology to sequence placental samples and concluded that there was a unique microbiota in the placenta, challenging people's long-standing perception of a “sterile uterus” (Aagaard et al., 2014). However, successive studies have reached conflicting conclusions. Some studies further confirmed the existence of placental microbiota and believed that placental microbiota played an important role in adverse pregnancy outcomes, such as preterm birth and fetal macrosomia (Collado et al., 2016; Prince et al., 2016; Parnell et al., 2017; Zheng et al., 2017a), but some studies which applied rigorous study design and data analysis negated the hypothesis of microbial colonization in the placenta of healthy pregnant women (De Goffau et al., 2019). Considering the low amount of microbiota in placenta and the difficulty of generating sequence libraries by 16S rRNA, metagenomic sequencing, or quantitative PCR methods were also used in some studies (Lauder et al., 2016; Prince et al., 2016). However, 16S rRNA gene amplicon sequencing has better sensitivity than metagenomic sequencing, especially in the microbiota study of human body sites involving human cells since no host DNA was sequenced (New and Brito, 2020). Therefore, inspired by de Goffau's experimental design (De Goffau et al., 2019), genomic DNA of the marine bacterium—“*Loktanella*” that did not exist in the human body was artificially introduced to achieve detectable sequencing of placenta, through which 16S rRNA sequencing was successfully constructed and completed in our study.

Our results showed most of the placental sample sequences came from artificially introduced genomic DNA of *Loktanella*. After excluding the exogenous bacterial DNA and non-16S DNA sequences, the number of reads in the placenta was low. Although it was found that the placenta and control shared 300 OTUs, there were still 571 OTUs only presented in the placenta samples, suggesting that the placenta might have its distinct bacteria. We assumed OTUs in placenta shared with the environmental control were from contamination and excluded from the analysis. The results showed that the main dominant bacterial phyla in the placental samples were *Firmicutes, Bacteroidetes, Actinobacteria*, and *Proteobacteria*, etc., which were consistent with previous studies (Aagaard et al., 2014; Bassols et al., 2016; Parnell et al., 2017). The dominant genera in the placenta after decontamination were mainly *Duncaniella, Alistipes, Odoribacter, Prevotella, Bacteroides, Bifidobacterium, Parasutterella, Limosilactobacillus, Ruminococcus, Anaerotignum, Helicobacter, Dialister*, etc., but most of the genera existed in only a few of the other samples. The existence of *Prevotella, Bacteroides, Bifidobacterium*, and *Ruminococcus* was also found in other studies of placenta microbiota (Aagaard et al., 2014; Zheng et al., 2017a; Gschwind et al., 2020; Tang et al., 2020). *Alistipes* and *Duncaniella* were the most abundant genera in our study samples. *Alistipes* was found primarily in the gut microbiota of healthy humans, and it was isolated from the blood stream, as well as abdominal, perirectal, and brain abscesses highlighting their potential opportunistic pathogenic roles in human diseases (Parker et al., 2020). There were few studies reporting *Alistipes* in human placenta samples, except that Ning Tang et al. reported *Alistipes* was in cord blood and placenta samples from GDM women (Tang et al., 2020). Studies have shown that the *Alistipes* spp. and *Bifidobacterium* spp. were lower in the gut microbiota with GDM women compared with healthy pregnant women (Hasain et al., 2020). The species of *Dialister* are mostly anaerobic or microaerobic Gram-negative bacteria, which are related to dental pulp infections, periodontitis, and other oral diseases. *D. pneumosintes* has been found in the placenta and amniotic fluid in previous studies (Morio et al., 2007). Moreover, genera *Odoribacter, Parasutterella*, and *Limosilactobacillu* were reported to predominantly preside in the gastrointestinal tract (Gomez-Arango et al., 2016; Antonson et al., 2020). A study found *Odoribacter sp*. from the gut microbiota was negatively correlated with blood pressure. It might help maintain lower systolic blood pressure in pregnant women through their production butyrate (Gomez-Arango et al., 2016). *Parasutterella* is a relatively new genus and mainly emerging from literatures of gut microbiome (Ju et al., 2019). Antonson et al. found that *Parasutterella excrementihominis* appears to be integral to inflammatory and metabolic dysregulation during prenatal stress (Antonson et al., 2020).

Our study found the women with GDM had a higher abundance of *Bacteroides, Bifidobacterium, Clostridium_XlVb, Duncaniella, Faecalibaculum, Harryflintia, Monoglobus, Ruminococcus*, and *Vampirovibrio* in placenta samples. Tang et al. reported a higher abundance of *Ruminococcus* in the placentas of GDM women, which was consistent with our results (Tang et al., 2020). A study investigated the placental microbiota of low birth weight in full-term neonates indicated a relative lower abundance of *Ruminococcus* (Zheng et al., 2015). Zheng et al. found that the proportion of *Proteobacteria* increased, and *Bacteroidetes* and *Firmicutes* decreased among women with GDM (Zheng et al., 2017b). However, our study found the genera belonging to *Firmicutes* (*Clostridium_XlVb, Faecalibaculum, Harryflintia, Monoglobus*, and *Ruminococcus*) were more abundant in placental samples of women with GDM. *Faecalibaculum, Harryflintia* (Petzoldt et al., 2016), and *Monoglobus* (Kim et al., 2019; Amat et al., 2021) were usually found in the gastrointestinal tract of animals. *Vampirovibrio chlorellavorus* is a predatory bacterium that can destroy a Chlorella culture in just a few days (Ganuza et al., 2016).

Our study identified eight genera with significantly higher abundance in the pregnant women with PROM, such as *Alistipes, Ihubacter, Bacteroides, Paraprevotella, Bifidobacterium, Ruminococcu, Harryflintia*, and *Parabacteroides*. Studies based on the method of microbial culture have found that the main pathogens derived from vaginal, placenta, and amniotic fluid of Chinese women with PROM were *Staphylococcus* and *Escherichia* (Zeng et al., 2014). *Lactobacillus* species in the vagina may increase the risk of PROM through microbial ascent to the uterus (Parris et al., 2021). The *Paraprevotella* genus is characterized by the production of succinic and acetic acid as major fermentation products. Only two species have been described from this genus and its potential effects on human health are still unknown (Morotomi et al., 2009). *Ihubacter* and *Parabacteroides* permanently preside in the human gut. *Parabacteroides* was found more enriched in the gut microbiota of GDM women (Hasain et al., 2020).

The detection of different species in the groups of adverse pregnancy outcomes might be led by higher species levels due to pathogenicity, which are easier to detect than potential “colonizing microorganisms.” An editorial in The Lancet Infectious Diseases emphasized that there was still considerable controversy in placental microbial research (The Lancet Infectious, 2018). Microbial studies with low biomass need to be cautious on the impact of possible contamination during research process. Future research should take the potential contamination into account that may exist in various ways and introducing more types of controls from potential contamination resources. In addition, it is suggested that the standardized operating specifications and procedures be established for the study of low-biomass microorganisms to obtain repeated results.

Our study introduced exogenous marine bacterial DNA that does not exist in the human body for detectable placental microbiota and used environmental samples as control to eliminate possible contaminations and identify the placental microbiota. The study findings indicated that the placenta contained low microbiota, and this was consistent with parts of existing studies at phylum level. But we found that many genera in the placenta were only present in a few samples, suggesting that human placenta may not have common microbiota. It was found that some of the genera in the soils or guts of animals also exist in the placenta, it may still have other contamination even though the contamination sequences of this study were eliminated through the study design of the environment control samples. In addition, our study considered all OTUs coexisting in the placenta and the environmental samples as contamination and excluded them from the analysis, which might lead to an underestimation of some real “existence” microbiota in the placenta. More rigorous study design is needed to avoid eliminating true placental bacteria in the future. Moreover, studies are also required to further determine the origin of placenta microbiota and its role in different adverse pregnancy outcomes with larger sample size. The mechanisms on such associations are needed to be further explored.

In conclusion, we found that there are low amounts of microbiota in human placenta. The predominant microbiota of the placenta might be varied between pregnant women with and without certain adverse pregnancy outcomes, such as GDM and PROM.

## Data Availability Statement

The datasets presented in this study can be found in online repositories. The names of the repository/repositories and accession number(s) can be found below: National Omics Data Encyclopedia (NODE) - OEP003119.

## Ethics Statement

The studies involving human participants were reviewed and approved by Ethics Committee of the School of Public Health, Fudan University. The patients/participants provided their written informed consent to participate in this study.

## Author Contributions

HJ conceived and obtained funding for this study. LS, FY, LZ, XC, and HJ coordinated and supervised study implementation and data collection. XL and WC participated in data collection. HZ and YW conducted lab test on the samples. XL and HZ conducted the data analysis. XL, YW, HJ, and HZ drafted the manuscript. XX and WC provided comments and revisions. All authors have approved to submit the manuscript for publication.

## Funding

This study was funded by the National Natural Science Foundation of China (81973057 and 82181220077), the Natural Science Foundation of Shanghai (19ZR1405900), the Shanghai Municipal Public Health Outstanding Discipline Leadership Program (GWV-10.2-XD10), and the Fifth Round of the Three-Year Public Health Action Plan of Shanghai (GWV-10.1-XK08).

## Conflict of Interest

The authors declare that the research was conducted in the absence of any commercial or financial relationships that could be construed as a potential conflict of interest.

## Publisher's Note

All claims expressed in this article are solely those of the authors and do not necessarily represent those of their affiliated organizations, or those of the publisher, the editors and the reviewers. Any product that may be evaluated in this article, or claim that may be made by its manufacturer, is not guaranteed or endorsed by the publisher.
